# Population Pharmacokinetics and Pharmacodynamic Target Attainment of Isavuconazole against *Aspergillus fumigatus* and *Aspergillus flavus* in Adult Patients with Invasive Fungal Diseases: Should Therapeutic Drug Monitoring for Isavuconazole Be Considered as Mandatory as for the Other Mold-Active Azoles?

**DOI:** 10.3390/pharmaceutics13122099

**Published:** 2021-12-06

**Authors:** Pier Giorgio Cojutti, Alessia Carnelutti, Davide Lazzarotto, Emanuela Sozio, Anna Candoni, Renato Fanin, Carlo Tascini, Federico Pea

**Affiliations:** 1 Institute of Clinical Pharmacology, Santa Maria della Misericordia University Hospital of Udine, ASUFC, 33100 Udine, Italy; piergiorgio.cojutti@aosp.bo.it; 2 SSD Clinical Pharmacology, IRCCS Azienda Ospedaliero Universitaria di Bologna, 40138 Bologna, Italy; 3 Infectious Diseases Clinic, Santa Maria della Misericordia University Hospital of Udine, ASUFC, 33100 Udine, Italy; alessia.carnelutti@asufc.sanita.fvg.it (A.C.); emanuela.sozio@asufc.sanita.fvg.it (E.S.); carlo.tascini@uniud.it (C.T.); 4 Division of Haematology, Santa Maria della Misericordia University Hospital of Udine, ASUFC, 33100 Udine, Italy; davide.lazzarotto@asufc.sanita.fvg.it (D.L.); anna.candoni@asufc.sanita.fvg.it (A.C.); renato.fanin@uniud.it (R.F.); 5 Department of Medicine, University of Udine, 33100 Udine, Italy; 6 Department of Medical and Surgical Sciences, Alma Mater Studiorum, University of Bologna, 40138 Bologna, Italy

**Keywords:** isavuconazole, hospitalized patients, therapeutic drug monitoring, Monte Carlo simulation

## Abstract

Isavuconazole is a newer broad-spectrum triazole approved for the treatment of invasive fungal disease. The objective of this study was to conduct a population pharmacokinetic and pharmacodynamic analysis of isavuconazole in a retrospective cohort of hospitalized patients. A nonlinear mixed-effect approach with Monte Carlo simulations was conducted to assess the probability of target attainment (PTA) of an area under the concentration–time curve (AUC_24 h_)/minimum inhibitory concentration (MIC) ratio of 33.4 (defined as efficacy threshold against *A. fumigatus* and *A. flavus*) associated with a maintenance dose (MD) of 100, 200 and 300 mg daily after loading. The cumulative fraction of response (CFR) against the EUCAST MIC distributions of *A. fumigatus* and *A. flavus* was calculated as well. The proportion of trough concentrations (C_trough_) exceeding a defined threshold of toxicity (>5.13 mg/L) was estimated. A total of 50 patients, with a median age of 61.5 years, provided 199 plasma isavuconazole concentrations. Invasive pulmonary aspergillosis was the prevalent type of infection and accounted for 80% (40/50) of cases. No clinical covariates were retained by the model. With the standard MD of 200 mg daily, CFRs were always ≥90% during the first two months of treatment. The risk of C_trough_ < 1.0 mg/L was around 1%, and that of C_trough_ > 5.13 mg/L was 27.7 and 39.2% at 28 and 60 days, respectively, due to isavuconazole accumulation over time. Our findings suggest that TDM for isavuconazole should not be considered as mandatory as for the other mold-active azoles voriconazole and posaconazole.

## 1. Introduction

Invasive fungal disease (IFD) represents one of the main causes of morbidity and mortality among immunocompromised patients [1]. Globally, the increased risk of developing an IFD is driven by the growing number of patients who receive intense immunosuppression after solid organ transplantation (SOT) and/or conditioning regimens for hematopoietic stem transplantation (HSCT) and/or immune-modulating agents for the treatment of various inflammatory conditions [2,3]. Invasive aspergillosis (IA) is a major cause of IFD, especially among immunocompromised patients, and *Aspergillus fumigatus* is by far the most frequent species involved, followed by *Aspergillus flavus*, *Aspergillus niger* and *Aspergillus terreus*. Despite available therapies, crude mortality rates from IFD remain high, with values ranging from 35.3% to as high as 80% in critically ill patients [4,5,6].

Triazoles have been the cornerstone of the management of IFD, with voriconazole being the first-line treatment option for IA and posaconazole being the main agent for prophylaxis of IA, and a salvage treatment of mucormycosis and other rare mold infections [7,8]. Isavuconazole is a second-generation triazole approved for the treatment of IA and, only in Europe, for the treatment of invasive mucormycosis in patients for whom amphotericin B is inappropriate [9]. From a pharmacokinetic point of view, isavuconazole is characterized by very high (>98%) plasma protein binding, long elimination half-life (80–130 h) and extensive hepatic metabolism by CYP3A4 [9]. It has some advantages compared to other triazoles. The pharmacokinetics are linear up to 600 mg daily, the risk of drug-drug interactions is low, the tolerability is better than with voriconazole and there are no issues in terms of oral bioavailability compared to posaconazole [9,10,11]. All these aspects lead to the clinical perception that routine therapeutic drug monitoring (TDM) of isavuconazole may not be necessary in most circumstances, also because clear evidence of the relationship between plasma concentrations and either efficacy or toxicity did not emerge in clinical trials [12]. This has been further supported by a recent report that showed a nearly identical distribution of the median isavuconazole concentration of 283 samples collected in the real-world clinical setting with that of 2458 samples collected in three phase III clinical trials [13].

Some studies showed that a certain degree of interindividual variability of isavuconazole concentrations may exist, spanning from nearly undetectable levels to plasma values higher than 10 mg/L, but no significant cofactors that may explain such a variability have been identified so far [13,14,15]. Although it could be expected that cotreatments with CYP3A4 modulators may play an important role in this regard, no previous pharmacokinetic model was informative so far.

The aim of this work was to conduct a population pharmacokinetic analysis of isavuconazole among hospitalized patients and a pharmacokinetic/pharmacodynamic study to assess the probability of target attainment with different dosing regimens of the pharmacodynamic threshold of efficacy against *A. fumigatus* and *A. flavus*.

## 2. Materials and Methods

### 2.1. Study Design

This was a monocentric, retrospective, observational study conducted among patients who were treated with isavuconazole because of IFD between September 2017 and November 2020 at the Azienda Sanitaria Universitaria Friuli Centrale of Udine, Italy.

Isavuconazole treatment was started with a loading dose of 200 mg every 8 h for two days, followed by a maintenance dose of 200 mg daily administered orally or intravenously, at the physician’s discretion. Patients underwent TDM of isavuconazole by assessing plasma C_trough_, which was determined approximately five minutes before the scheduled daily dose, and, whenever possible, also plasma C_peak_, which was determined 2 h after oral administration or 0.5 h after a 1 h intravenous infusion. TDM was assessed firstly after at least 72 h from starting therapy and subsequently reassessed at the discretion of the attending physician. At our hospital, the reference range of isavuconazole C_trough_ was set between 1 and 5.13 mg/L. This was arbitrarily set on the basis of the findings of a real-life study [13], which proposed a lower threshold of 1 mg/L in order to achieve plasma levels above the EUCAST clinical breakpoint of *A. fumigatus* and *A. flavus* [16], and of the findings of Furfaro et al. [14], who identified at ROC analysis an upper threshold of 5.13 mg/L as associated with the occurrence of some mild adverse events during long-term treatment, such as nausea, anorexia and fatigue.

Blood samples for isavuconazole TDM were immediately delivered to the Institute of Clinical Pharmacology, where they were centrifuged to obtain serum. Isavuconazole serum concentrations were estimated by means of a validated liquid chromatography–tandem mass spectrometry analytic method [17]. Precision and accuracy were assessed by replicate analysis of quality control samples against calibration standards. Intra- and interassay coefficients of variation were always <10%. The LOQ was 0.11 mg/L, and the assay was linear in the range of 0.1–10 mg/L.

The following demographic and clinical data were retrieved from each patient’s medical record: age, gender, weight, height, type and site of infection and microbiological isolate. Patient comedications, along with serum albumin, total bilirubin, ALT, AST and γ-GT concentrations, were recorded at each TDM assessment. Drug-induced liver injury was defined as mild, moderate, severe or fatal according to the EASL Clinical Practice Guidelines for drug-induced liver injury [18]. Drug cotreatments were accurately reviewed by a clinical pharmacologist with the intent of assessing the eventual influence on isavuconazole clearance by CYP3A4 inhibitors and/or inducers. CYP3A4 inhibitors and/or inducers were classified as strong, moderate and mild according to the FDA [19] and to the DrugBank database [20]. Patient outcome at end of therapy was defined as successful (in case of clinical, radiological and/or mycological resolution or improvement of infection) or failed according to [21].

### 2.2. Population Pharmacokinetic Modeling

Population pharmacokinetics were conducted using the nonparametric adaptive grid approach (NPAG) and the algebraic model solver included in the Pmetrics package (Version 1.5.0, Laboratory of Applied Pharmacokinetics and Bioinformatics, Los Angeles, CA, USA) of R [22]. One- and two-compartment models with first-order absorption and first-order elimination from the central compartment were developed. Maximum a posteriori (MAP)-Bayesian estimates of isavuconazole clearance (CL) and volume of distribution (V) were determined in each patient.

Influence of covariates was assessed by including the biologically plausible clinical covariates into the basic model. Covariate analysis was conducted by means of the Pmetrics function “PMstep” and by linear regression between the median MAP-Bayesian estimates of isavuconazole pharmacokinetic parameters and each tested covariate.

Model comparison was performed by calculating the objective function value (OFV) and the Akaike information criterion (AIC) value. An improvement between the two competitive models was considered statistically significant when the decrease in the OFV was at least 3.84 points. For both the population predictions and the individual predictions, model fit was assessed by evaluating the coefficient of determination of the linear regression of the observed versus predicted concentration plot. Internal model validation was performed by means of a visual predictive check (VPC) with *n* = 1000 simulations. The normalized prediction distribution errors (NPDE) were also calculated. The VPC plot compares the observed concentrations with model-predicted concentration-time profiles. A model is reliable when ≥95% of the observed concentrations reside within the 95% CI of the model prediction. Assay laboratory error was estimated by means of the interday variability assay data. A first-order polynomial regression was used between drug concentrations and the standard deviation of the observations (C0 = 0.006, C1 = 0.189). Extra process noise was captured with a gamma (G) model (G = 2).

### 2.3. Monte Carlo Simulation Analysis and Probability of Target Attainment

One-thousand-subject Monte Carlo simulations were conducted using Pmetrics to estimate the isavuconazole exposure achievable, in terms of the 24 h area under the concentration–time curve (AUC_24 h_) and C_trough_, with a loading dose of 200 mg every 8 h for two days, followed by three different incremental maintenance doses of 100, 200 or 300 mg daily. Isavuconazole C_trough_ and AUC_24 h_ were assessed at the end of loading and then on Days 7, 14, 21, 28 and 60.

The probability of target attainment (PTA) of an AUC_24 h_/MIC > 33.4 was calculated at each of these time points. This pharmacodynamic threshold is that suggested by EUCAST and is based on the EUCAST MIC method [16]. It is based on an immunocompetent murine model, where it was shown to be associated with a 90% survival rate against disseminated aspergillosis caused by wild-type and azole-resistant *A. fumigatus* [23].

The cumulative fraction of response (CFR) achievable against the EUCAST MIC distribution of *A. fumigatus* and *A. flavus* with the three tested dosing regimens was calculated [24], and a percentage ≥90% was considered as optimal.

### 2.4. Statistics

Data were presented as median and interquartile range (IQR) for continuous variables or as count and percentage for categorical variables in the descriptive statistics. Univariate and multivariate linear mixed-effect models (with a random effect to account for correlation among repeated measurements within the same subject) were performed to identify clinical independent predictors of isavuconazole C_trough_. All variables that were associated with a *p* ≤ 0.20 at univariate analysis were subsequently included in the multivariate model. All statistical and graphical analyses were performed with R version 3.4.4 (R Foundation for Statistical Computing, Vienna, Austria). Data were considered statistically significant when *p* < 0.05.

### 2.5. Ethics

This study was approved by the Ethics Review Board of the Friuli-Venezia-Giulia Region (Protocol Number: 0017855/CEUR, approved: 29 May 2020). Written informed consent was waived due to the retrospective and observational nature of this investigation.

## 3. Results

A total of 50 consecutive patients were included in this study. Patient demographic and clinical characteristics are summarized in Table 1.

Males accounted for 62% (31/50) of the population. Median (IQR) age and body weight were 61.5 (51.3–72.0) years and 65 (55.5–71.5) kg, respectively. Invasive pulmonary aspergillosis represented the most frequent type of IFD and accounted for 80% (40/50) of cases. Four patients (8%, 4/50) had non-Aspergillus IFD, whereas five patients (10%, 5/50) had probable IFD. Forty-five patients (90%, 45/50) received isavuconazole as first-line therapy, whereas the other five (8%, 4/50) were switched to isavuconazole after previous therapy with voriconazole or posaconazole.

Isavuconazole was administered mainly orally (76%, 38/50) for a median (IQR) duration of treatment of 48 (19–91) days. No patient was cotreated with strong CYP3A4 inhibitors and/or inducers, whereas 10 out of 50 (20%, 10/50) were cotreated with mild or moderate CYP3A4 inhibitors (namely loperamide, haloperidol, venetoclax, cyclosporine, letermovir and sorafenib).

**Figure 1:**
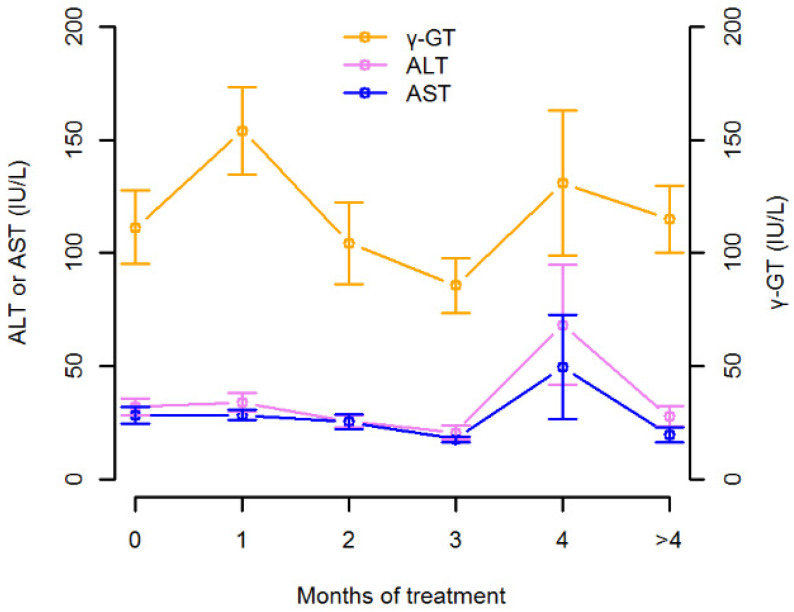
Temporal trend of alanine-aminotransferase (ALT), aspartate-aminotransferase (AST) and gamma-glutamyltransferase (γ-GT) concentrations in relation to months of treatment. Symbols and whiskers represent mean and standard errors of the enzyme concentrations at each month.

Median (min–max) range of alanine-aminotransferase (ALT), aspartate-aminotransferase (AST) and gamma-glutamyltransferase (γ-GT) was 21.0 (3.0–261.0) UI/L, 20.0 (5.0–249.0) UI/L and 70.0 (12.0–642.0) IU/L, respectively. Figure 1 shows the temporal trend of ALT, AST and γ-GT concentrations in relation to months of treatment. No patient experienced signs and/or symptoms of isavuconazole toxicity during therapy. In one patient, an intercurrent mild hepatotoxicity during isavuconazole treatment (ALT raised up to 261 IU/L, total bilirubin was 0.17 mg/dL) was observed, but it resolved spontaneously within three days with no need of isavuconazole withdrawal.

Among the forty-seven patients who completed isavuconazole treatment, clinical outcome was successful in 68.1% (32/47) of cases, failure occurred in 19.2% (9/47) of cases, lack of significant improvement with isavuconazole treatment was observed in 6.4% (3/47), whereas 6.4% (3/47) died because of progression of underlying oncohematological disease.

### 3.1. Isavuconazole Measurements and Regression Analysis

Overall, 199 isavuconazole concentrations ((175 trough concentration (C_trough_) and 24 peak concentrations (C_peak_)) were included in the pharmacokinetic analysis. Of the 175 C_trough_, 2 (1.1%) were <1.0 mg/L, and 50 (28.6%) were >5.13 mg/L. The clinical variables tested at univariate and multivariate mixed-effect regression analysis as potential covariates of isavuconazole C_trough_ were age, weight, gender, dose/kg, days from starting therapy, albumin, total bilirubin, ALT, AST, γ-GT and cotreatments with mild/moderate CYP3A4 inhibitors (Table 2). Among these, cotreatment with mild/moderate CYP3A4 inhibitors was significantly associated with a more than 2-fold increase in isavuconazole C_trough_. Conversely, patient age was associated with a 3.7% reduction in C_trough_.

### 3.2. Population Pharmacokinetic Modeling

**Figure 2:**
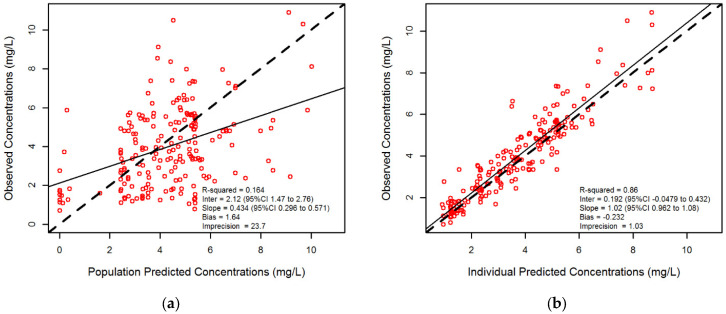
Diagnostic plot of the population pharmacokinetic model. (**a**) observed plasma concentrations versus population predicted concentrations; (**b**) observed plasma concentrations versus individual predicted concentrations performed after the individual Bayesian optimization step.

The population pharmacokinetic model that best described isavuconazole concentration-time data was a two-compartment linear model with first-order input (for orally administered doses), first-order clearance from the central compartment and first-order intercompartmental clearance (Q). Compared to a one-compartment model, it showed lower OFV (549 vs. 654) and AIC (563 vs. 664.3). Patient age and cotreatments with CYP3A4 inhibitors were also tested as potential covariates on isavuconazole clearance, but they did not improve model fit. The relationship between isavuconazole observed versus predicted concentrations on a population level (R^2^ = 0.16; bias = 1.64; imprecision = 23.7) and after Bayesian estimation (R^2^ = 0.86; bias = −0.23; imprecision = 1.03) are shown in Figure 2.

**Figure 3:**
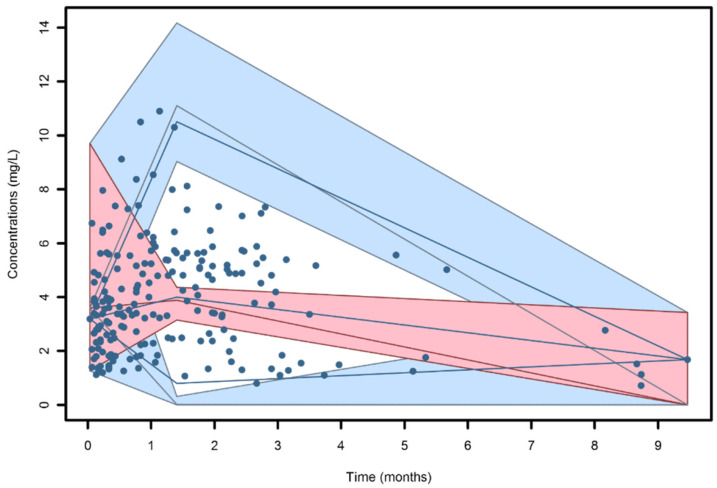
Visual predictive check (VPC) of isavuconazole plasma concentration versus time for the final model. The colored areas represent the 95% prediction intervals calculated on the 2.5th and 97.5th percentiles (blue areas) and on the 50th percentile (pink area) of simulated data. Continuous blue lines correspond to the 2.5th, 50th and 97.5th percentiles of the observed data.

The VPC plot (Figure 3) showed a good predictive performance of the model, as the 2.5th, 50th and 97.5th percentiles of the observed data were within the colored areas. Residuals were normally distributed (*p* = 0.638 at the Shapiro-Wilk test for NPDE) and were symmetrical around zero (*p* = 0.339 at the symmetry test for NPDE).

Table 3 shows the population estimates of the individual pharmacokinetic parameters for the final model.

### 3.3. Monte Carlo Simulation and Probability of Target Attainment

**Figure 4:**
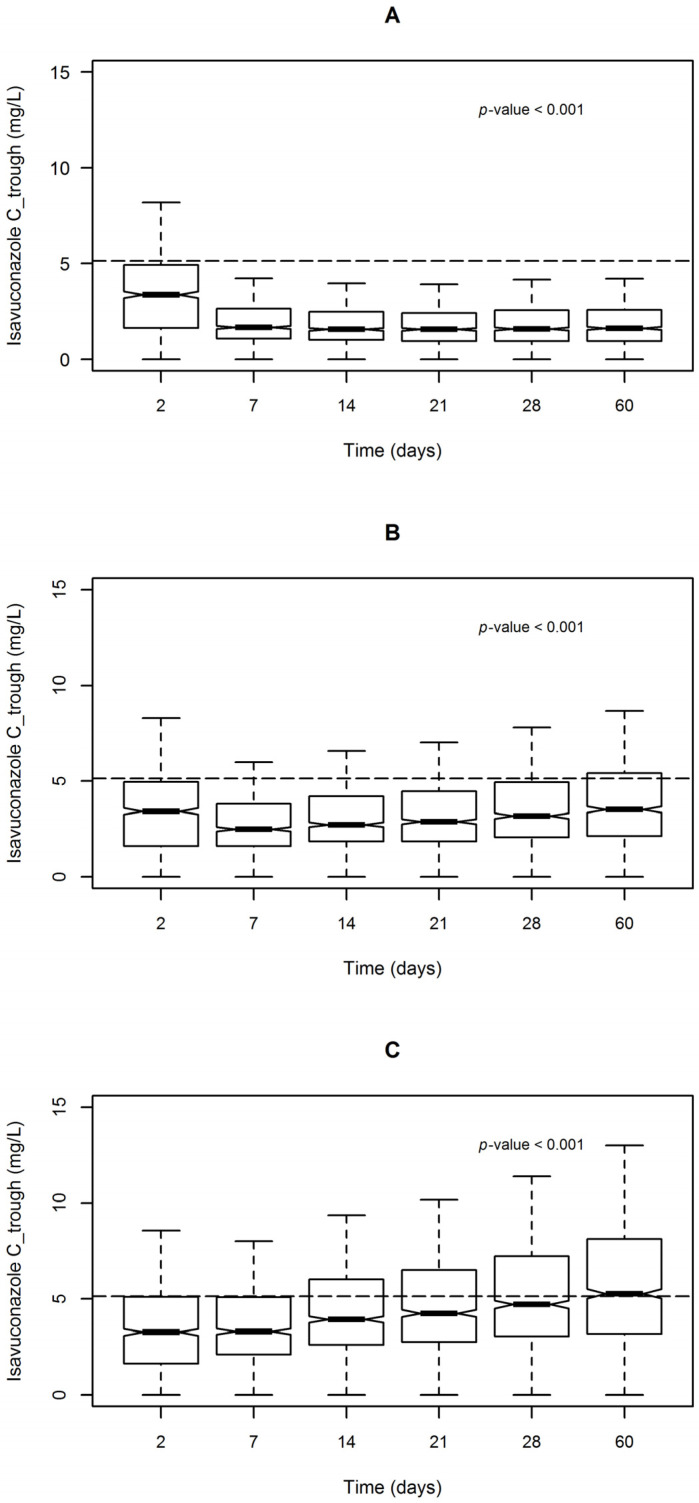
Box (median and 25–75th percentiles) and whiskers (5–95th percentiles) plot of isavuconazole trough concentrations (C_trough_) following administration of a loading dose of 200 mg every 8 h followed by a maintenance dose of 100 mg daily (**A**), 200 mg daily (**B**) or 300 mg daily (**C**). The dashed line identifies the isavuconazole toxicity threshold (C_trough_ > 5.13 mg/L). A value of *p* < 0.001 was obtained in Kruskal-Wallis test.

Figure 4 summarizes the distribution of simulated isavuconazole C_trough_ in relation to the duration of treatment associated with the three dosing regimens. A statistically significant drug accumulation was observed over time with all of these regimens (*p* < 0.001).

The frequencies of distribution of simulated isavuconazole C_trough_ < 1 mg/L, between 1–5.13 mg/L and >5.13 mg/L associated with each dosing regimen over time, are reported in Table 4.

At the end of the loading period, the vast majority of C_trough_ estimates were ≥1 mg/L (98.3%). During the maintenance period, the standard dosage of 200 mg granted the best exposure in terms of reference range with a very low probability of underexposure (<1 mg/L in less than 5% of cases), associated with a progressively increasing, but acceptable, probability of overexposure over treatment (>5.13 mg/L in 17.8, 27.7 and 39.2% of cases on Days 14, 28 and 60, respectively). Conversely, decreasing dosage to 100 mg may expose to a higher risk of underexposure (<1 mg/L in more than 15% of cases in the first 2 weeks and more than 10% of cases on Day 60). Increasing dosing up to 300 mg resulted in an unacceptably high probability of overexposure over treatment (>5.13 mg/L in 39.2, 53.0 and 73.2% of cases on Days 14, 28 and 60, respectively).

Optimal CFRs of an AUC_24 h_/MIC > 33.4, which is the pharmacodynamic index of efficacy based on EUCAST clinical breakpoint, against the EUCAST MIC distribution of *A. fumigatus* and *A. flavus* were achievable during the whole treatment period only when considering the standard 200 mg daily maintenance dose (Table 5).

**Figure 5:**
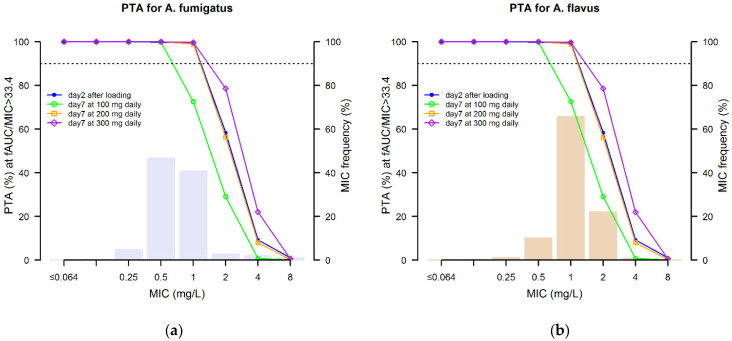
Probability of target attainment (PTA) of AUC_24 h_/MIC > 33.4 after loading and on Day 7 associated with isavuconazole maintenance doses of 100, 200 and 300 mg daily (solid lines) against the EUCAST MIC distribution (histograms) of *A. fumigatus* (**a**) and *A. flavus* (**b**). Horizontal dotted lines identify the threshold for optimal PTA (90%).

Figure 5 shows the distribution of the PTAs achievable after loading and on Day 7 with the MD of 100, 200 and 300 mg daily in relation to the EUCAST MIC distribution of *A. fumigatus* and *A. flavus*. The standard 200 mg daily dose achieved optimal PTAs at the clinical breakpoint of 1 mg/L against both *A. fumigatus* and *A. flavus*.

## 4. Discussion

To the best of our knowledge, this is the first population pharmacokinetic study of isavuconazole conducted in real-world hospitalized patients who received isavuconazole for the treatment of IFD.

Our study confirmed that isavuconazole pharmacokinetic behavior may be best described by a two-compartment model, similar to what was previously observed in three large-population pharmacokinetic studies based on data coming from phase I and III clinical trials [25,26,27] and in another one carried out among 96 solid organ transplanted (SOT) patients who received isavuconazole for prophylaxis [28]. The wide median volume of distribution (488.51 L) was consistent with previously reported values [26,27,29]. The median CL estimate (1.52 L/h) was somewhat lower than that reported in clinical trials (2.2–2.5 L/h) [26,27] and consistently lower than that observed in SOT patients (4.28 L/h). The pharmacokinetic model was reliable in predicting observed concentrations (with good precision and R^2^ and low bias), and the interindividual variability on CL (64%) was in line with that reported by previous analysis (43–63%) [30].

The mixed-effect linear regression analysis showed that both age and cotreatments with mild/moderate CYP3A4 inhibitors affected isavuconazole C_trough_. However, none of these covariates improved the fitting of the population model. Although isavuconazole is a substrate of CYP3A4 [31], and it is expected that modulators of CYP3A4 activity would affect isavuconazole CL, in our model, we were unable to ascribe any interindividual pharmacokinetic variability of isavuconazole CL to any cotreatments with CYP3A4 inhibitors. This could be due to the fact that our patients received only mild to moderate CYP3A4 inhibitors. Perhaps strong inhibitors, such as ketoconazole and high-dose ritonavir, and/or strong inducers, such as rifampicin and barbiturates, could have affected more significantly isavuconazole CL [32,33]. Other studies investigated the effect of possible covariates on isavuconazole CL and showed conflicting results, with some reporting gender [28] or liver function [25] as significant covariates and others observing no effect [26,27].

Monte Carlo simulations showed an increase in C_trough_ over time, suggesting drug accumulation during treatment. This is in agreement with what was observed in a preliminary multiple-dose study conducted in 32 healthy volunteers, which showed a plasma drug accumulation ratio between start and end of therapy in the range of 3.8–5.2 and comparable after both IV and oral administration [34]. A similar finding was also observed in a retrospective analysis of isavuconazole C_trough_ (*n* = 264 samples) from 19 patients who underwent TDM in a tertiary-care Italian hospital [14]. A linear mixed model was used to assess the longitudinal concentrations of isavuconazole over time and showed a linear increase of 0.032 mg/L for each day of treatment [14]. More recently, two other retrospective analyses confirmed that drug accumulation may protract over several months with ongoing therapy in some patients [35,36].

Importantly, Monte Carlo simulations showed that the use of a standard 200 mg maintenance daily dosage after loading may ensure effective exposure in almost all of the patients, although during long-term treatment, some accumulation may occur, with a potential overexposure in up to one-third of patients. In this regard, it should not be overlooked that in the serial monitoring of isavuconazole concentrations reported by Furfaro et al., high C_trough_ were associated mainly to mild adverse events, such as nausea, hyporexia and fatigue, but not with a hepatotoxicity risk [14]. Median isavuconazole concentrations on days when side effects occurred were significantly higher compared to that observed when side effects were not present (6.65 vs. 4.09 mg/L, *p* < 0.001), and a cut-off value for toxicity of 5.13 mg/L was identified at ROC analysis. Importantly, the side effects were more likely to occur during prolonged treatment (median duration of therapy of 134 days (range: 67–250 days) in those with side effects versus 79 days (range: 2–230 days) in those with no side effects) [14]. Indeed, there is only one case report suggesting that very high isavuconazole levels >10 mg/L could have been associated with the occurrence of major toxicity, namely hepatic veno-occlusive disease, in a 19-year old male with AML relapsed after haploidentical transplantation [15]. Consequently, clinicians may be reassured that even quite high isavuconazole C_trough_ seem not to be associated with major adverse events and/or toxicity.

Overall, these findings suggest that the standard 200 mg maintenance dose could be appropriate in dealing with IFD in most patients, also considering that it may ensure optimal CFRs against both *A. fumigatus* and *A. flavus* over the first two months of treatment. This implies that TDM should not be considered for isavuconazole as mandatory as for the other mold-active azoles voriconazole and posaconazole, whose pharmacokinetic behavior is largely unpredictable.

Current guidelines [37] do not recommend TDM for isavuconazole. Indeed, our data support this contention, even if TDM could represent a helpful tool in some specific cases. This is in agreement with the recent findings of a retrospective comparative study of TDM of isavuconazole (*n* = 273 samples from 35 patients) vs. voriconazole (*n* = 1424 samples from 283 patients) from Denmark [30], which confirmed that isavuconazole is much easier to target compared to voriconazole and concluded that the role of TDM for isavuconazole is not so compelling.

We recognize that this study has some limitations. The retrospective design, the population case mix and the limited number of patients must be acknowledged, and this may limit the generalizability of our findings. On the other hand, the Monte Carlo simulation and the accurate PK/PD analysis of the proportion of simulated concentrations reaching the target during treatment are strengths that may represent innovative information. Further prospective large-scale studies are warranted to confirm our findings.

## 5. Conclusions

In conclusion, we developed for the first time a population pharmacokinetic model for isavuconazole from real-life hospitalized patients and we showed with Monte Carlo simulations that the standard 200 mg daily maintenance dose is suitable for attaining optimal CFRs against *A. fumigatus* and *A. flavus* during the first two months of treatment. As the pharmacokinetic of isavuconazole is scarcely affected by clinical covariates and cotreatments, TDM for this antifungal may be less indicated than for the other azoles.

## Figures and Tables

**Table 1:** Characteristics of the study population (*n* = 50).

Variable	Median or Count	Range or %
Age (years)	61.5	51.3–72.0
Gender (male/female)	31/19	62/38
Body weight (kg)	65.0	55.5–71.5
Albumin (g/L)	35.0	28.4–40.0
Total bilirubin (mg/dL)	0.28	0.2–0.4
Gamma-glutamyltransferase (IU/L)	70.0	42.0–173.0
Alanine-aminotransferase (IU/L)	21.0	15.0–38.0
Aspartate-aminotransferase (IU/L)	20.0	15.0–31.0
Type of infections		
Invasive pulmonary aspergillosis	40	80.0
Invasive fusariosis	2	4.0
Cerebral mucormycosis	1	2.0
Scedosporium osteomyelitis	1	2.0
Aspergillus brain abscess	1	2.0
Invasive fungal disease, not specified	5	10.0
Underlying disease		
Oncohematological malignancy	25	50.0
Nosocomial pneumonia	11	22.0
Immunosuppression°	9	18.0
Other	5	10.0
Isavuconazole treatment		
First-line or switch from other azoles	45/5	90/10
Dose (mg)	200	200–200
Total number of C_trough_	175	
C_trough_ (mg/L)	3.68	2.07–5.38
Total number of C_peak_	24	
C_peak_ (mg/L)	4.67	3.78–5.96
Number of TDM instances	2.0	1.0–4.0
Treatment duration (days) *	48.0	19.0–91.0
Clinical outcome at end of treatment *		
Successful treatment	32	68.1
Treatment failure	12	25.5
Dead for other reasons	3	6.4

C_trough_, isavuconazole trough (minimum) concentration; C_peak_, isavuconazole peak (maximum) concentration. * available only for patients who completed treatment course (*n* = 47). Immunosuppression included: solid organ transplant, solid malignant neoplasms and rheumatological diseases.

**Table 2:** Univariate and multivariate mixed-effect linear regression analysis of clinical variables associated with isavuconazole C_trough_.

	Univariate Analysis		Multivariate Analysis	
Variables	Unstandardized β-Coefficient (95% CI)	*p*-Value	Unstandardized β-Coefficient (95% CI)	*p*-Value
Age (years)	0.037 (0.066–0.007)	0.022	0.037 (0.061−0.013)	<0.001
Weight (kg)	−0.029 (0.006−0.064)	0.106		
Gender (male vs. female)	0.099 (6.986−6.788)	0.977		
Dose/kg daily (mg/kg)	0.815 (1.164–0.466)	0.010	0.402 (0.819−0.016)	0.067
Days from starting therapy (days)	0.001 (0.007−0.005)	0.747		
Albumin (g/L)	0.034 (0.087−0.019)	0.214		
Total bilirubin (mg/dL)	−0.346 (0.034−0.726)	0.078		
ALT (IU/L)	−0.001 (0.007−0.009)	0.730		
AST (IU/L)	−0.008 (-0.002–0.004)	0.230		
γ-GT (IU/L)	0.003 (0.005–0.001)	0.022	−0.0004 (0.002−0.002)	0.751
Cotreatment with CYP3A4 inhibitors	2.39 (3.337–1.443)	0.039	2.154 (3.248–1.060)	0.018

**Table 3:** Parameter estimates for the final population pharmacokinetic model of isavuconazole.

	CL (L/h)	Ka (h^−1^)	Fos (%)	Q (L/h)	V (L)	Vp (L)
Mean	1.52	22.64	0.95	16.78	89.50	735.24
SD	0.97	3.54	0.07	18.35	42.38	633.89
CV (%)	64.03	15.66	7.42	109.37	47.35	86.22
Median	1.33	22.64	1.00	5.08	102.58	385.93

CL, total body clearance; Ka, first-order transfer rate constant of absorption; Fos, oral bioavailability; Q, intercompartmental clearance; V, volume of distribution of the central compartment; Vp, volume of distribution of the peripheral compartment.

**Table 4:** Probability of achievement of isavuconazole trough concentrations (C_trough_) < 1.0, 1.0–5.13, >5.13 mg/L on Day 2 after LD, and on Days 7, 14, 21, 28 and 60 after MD of 100, 200 or 300 mg daily, as predicted by Monte Carlo simulation.

	LD	MD of 100 mg Daily					MD of 200 mg Daily					MD of 300 mg Daily				
Isavuconazole C_trough_ (mg/L)	Day 2	Day 7	Day 14	Day 21	Day 28	Day 60	Day 7	Day 14	Day 21	Day 28	Day 60	Day 7	Day 14	Day 21	Day 28	Day 60
<1.0	1.7	21.7	16.4	12.9	12.0	11.7	4.1	1.8	1.3	1.0	1.1	0.8	0.2	0.2	0.1	0.1
1.0–5.13	85.2	76.4	81.5	84.6	83.8	81.1	84.3	80.4	73.6	71.3	59.7	76.9	60.6	48.6	46.9	26.6
>5.13	13.1	1.9	2.1	2.5	4.2	7.2	11.6	17.8	25.1	27.7	39.2	22.3	39.2	51.2	53.0	73.2

LD, loading dose (200 mg q8 h for 48 h); MD, maintenance dose.

**Table 5:** Cumulative fraction of response (CFR) of three isavuconazole dosing regimens against EUCAST MIC distribution of *Aspergillus fumigatus* (*n* = 426) and *Aspergillus flavus* (*n* = 434).

	*Aspergillus fumigatus*						*Aspergillus flavus*					
Isavuconazole Dosing Regimens	Day 2	Day 7	Day 14	Day 21	Day 28	Day 60	Day 2	Day 7	Day 14	Day 21	Day 28	Day 60
LD + MD of 100 mg daily	94.7	82.7	84.5	88.8	89.9	89.5	90.0	65.6	67.9	75.4	76.2	78.3
LD + MD of 200 mg daily	94.7	94.5	95.4	95.8	96.2	96.6	90.0	90.2	92.4	94.4	96.6	96.8
LD + MD of 300 mg daily	94.7	95.8	96.7	97.2	97.3	97.9	90.0	94.5	98.1	98.9	98.9	99.1

LD, loading dose (200 mg q8 h for 48 h); MD, maintenance dose.

## Data Availability

The data presented in this study are available on request from the corresponding author. The data are not publicly available due to privacy concerns.
